# High-Speed Full-Color Polarized Light Imaging of Collagen Using a Polarization Camera

**DOI:** 10.3390/bioengineering12070720

**Published:** 2025-06-30

**Authors:** Bin Yang, Neil Nayyar, Billy Sanchez

**Affiliations:** Department of Biomedical Engineering, Duquesne University, Pittsburgh, PA 15282, USA; nayyarn@duq.edu (N.N.); sanchezb@duq.edu (B.S.)

**Keywords:** polarized light imaging, polarization-sensitive camera, collagen characterization, fiber orientation, high-speed imaging

## Abstract

Polarized light imaging (PLI) has been effective in visualizing and quantifying collagen content. Collagen-specific data are often overlaid over the tissue image for visualization. However, such contextual tissue images are typically in grayscale and lack important color information, limiting the usefulness of PLI in imaging the stained histology slides and for surgical guidance. The objective of this study was to develop a robust and easy-to-implement PLI technique to capture both true color and birefringent collagen data, and we call it ColorPOL. ColorPOL uses only one polarization-sensitive camera to capture information at 75 frames per second. The true color images were synthesized from individual RGB images, and collagen-specific information (fiber orientation and retardance) was derived from the green channel image. We implemented ColorPOL in transmission mode on an upright microscope and in reflection mode for wide-field thick tissue imaging. The color images in both implementations provided valuable color tissue context that facilitated the identification and localization of collagen content. Additionally, we demonstrated that in reflection mode, the high imaging speed enabled us to record and visualize continuous deformations of the collagenous tissues (tendons, sciatic nerves, and blood vessels) overlaid on the processed collagen-specific information. Robust performance and flexible configuration will make ColorPOL a valuable tool in basic research and translational applications.

## 1. Introduction

Collagen is a structural protein that is the main building block of the load-bearing connective tissues, including tendons, heart valves, skin, and cornea [1,2,3,4,5]. Collagen fiber organization is tissue-dependent, ranging from highly anisotropic (e.g., tendon) to nearly isotropic (e.g., sclera), to provide specific mechanical support and physiological functions [5]. Visualizing collagen distribution and quantifying their fiber orientation, both with and without external load, are central to the biomechanics and pathophysiology of collagenous tissues [6,7]. Collagen fibers are birefringent due to their highly ordered and anisotropic structure, which makes polarized light-based imaging technologies effective tools for interrogating their microstructure [8,9]. Polarized light microscopy/imaging has been widely used for visualizing collagen content. In recent years, polarization-sensitive optical coherence tomography (PS-OCT) [10,11] and polarization-sensitive second harmonic generation (PS-SHG) [12,13] microscopy were developed to study the 3D organization of collagen content.

Collagen-specific information is often visualized as an overlay on the tissue images. If the tissue itself is mostly colorless, it might be sufficient to augment the collagen information on a grayscale image [6]. Such grayscale images can be conveniently generated from polarization images without the need for an additional camera. However, if collagen content is surrounded by tissues showing complex natural (e.g., in a surgical field) or artificial (e.g., stained histology slides) colors, using a grayscale image would inevitably result in the loss of critical tissue information. To overcome such limitations, polarized light imaging systems with a dual-camera setup have been developed with a color camera dedicated to capturing color images and a grayscale camera for capturing polarization images [14,15]. These dual-camera techniques have been demonstrated to be useful in studying histology slides and for surgical navigation and guidance. These systems, however, typically require more sophisticated instrument configuration and control, as well as more complex data processing and visualization pipelines [16]. Final visualization quality is highly dependent on the precise alignment and registration of images from the two cameras. Recently, polychromatic/instant polarization microscopy has been developed to acquire both natural color and color-coded collagen information simultaneously [17,18,19], enabling real-time visualization without the need for additional computation. However, these imaging techniques are mostly suitable for imaging thin-tissue sections [20]. Their applicability to thick tissues in reflection mode has not been demonstrated, limiting their applications.

Recognizing the challenges and limitations of existing polarized light imaging, the objective of this study was to develop a polarized imaging technique that could capture both color and collagen information at high speed and be implemented in both transmission and reflection modes, using only one polarization-sensitive camera. We term our imaging technique as ColorPOL. The rest of the paper will describe in detail the system configuration, data processing and visualization pipeline, and the results obtained in both transmission and reflection modes.

## 2. Materials and Methods

### 2.1. Imaging System Development

We developed two variants of the ColorPOL imaging system to demonstrate its capabilities for microscopy (transmission) and wide-field (reflection) imaging applications. These two imaging setups share similar configurations of illumination and imaging sub-systems.

**Figure 1:**
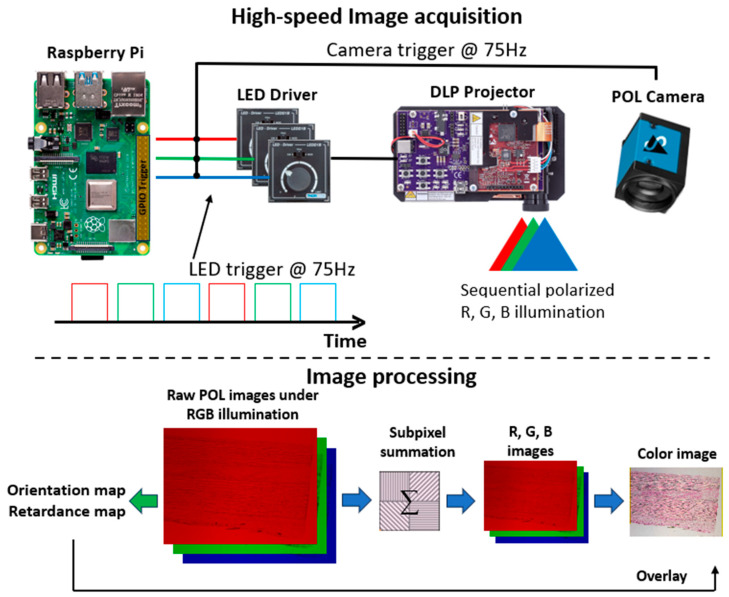
Upper panel: Core ColorPOL imaging system configuration. Illumination and image acquisition are synchronized through a trigger signal generated with a Raspberry Pi at 75 Hz. Lower panel: Pipeline of image processing and visualization. Color images are generated using polarization images under R, G, and B illumination. The polarization image under green illumination is used to derive the fiber orientation map and retardance map, which are overlaid on the corresponding color image.

Illumination sub-system: We adopted a digital light processing (DLP) projector (DLP3010EVM-LC, TI, Dallas, TX, USA) as the illumination source for both systems (Upper panel in Figure 1). A DLP projector generates highly uniform illumination over a large field, which is particularly useful in wide-field imaging. The light engine of the projector consists of red, green, and blue LEDs. The projector, however, does not natively support rapidly changing the illumination colors during operation. To circumvent this limitation, we developed a separate light engine control system based on a Raspberry Pi and dedicated LED drivers (LEDD1B, Thorlabs, Newton, NJ, USA). The brightness of each LED can be adjusted, and the LEDs can be individually triggered via a trigger signal generated on the Raspberry Pi. LEDs were triggered continuously and sequentially at 75 Hz. A camera trigger was sent after each LED trigger to acquire an image under the current illumination. A circular polarizer (88-097, Edmund Optics, Barrington, NJ, USA) was used to generate circularly polarized illumination.

Imaging sub-system: A polarization-sensitive camera (DZK 33UX250, Imaging Source, Charlotte, NC, USA) was used for acquiring images. The camera sensor features micropolarizers in front of individual pixels. The micropolarizers are grouped into a 2 × 2 configuration with 4 linear polarization states (0,45, 90, and 135 degrees) [21]. The camera allows for acquiring images of 4 polarization states in a single snapshot, a feature that enables rapid ColorPOL imaging. The camera was synchronized with the illumination to achieve high-speed image acquisition at 75 frames/second (FPS).

**Figure 2:**
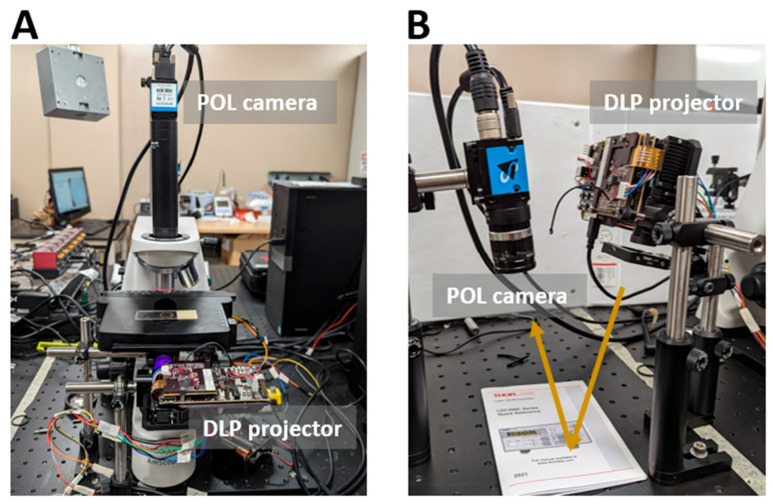
System configurations of (**A**) ColorPOL microscope in transmission mode and (**B**) ColorPOL wide-field imaging in reflection mode.

Microscope configuration (transmission mode): We removed the built-in condenser lens and ocular head of an upright microscope (Figure 2A), enabling the integration of ColorPOL components. A 35 mm biconvex lens was placed right before the projector lens to condense the illumination before it was reflected upwards by a 45-degree mirror toward the sample stage. A circular polarizer was placed above the 45-degree mirror to generate circularly polarized illumination. A 4x objective lens was used for imaging samples.

Wide-field imaging configuration (reflection mode): In this configuration, the projector was slightly angled (around 15 degrees) to provide a uniform illumination that covered the entire field (approximately 7 cm × 11 cm) (Figure 2B). A circular polarizer was placed directly before the projection lens. We used a 35 mm C-mount lens (HF35HA, Fujinon, Tokyo, Japan) for image acquisition. The aperture of the lens was adjusted to ensure a sufficient depth of field. During testing, the imaging setup continuously acquired images at 75 FPS until it was stopped.

Because the color image was reconstructed from red, green, and blue channel images in post-processing, we adjusted the LEDs individually to maximize the illumination intensity without causing saturation to leverage the dynamic range of the sensor. We developed a LabVIEW program to facilitate intensity adjustment by displaying the real-time intensity histogram of each channel. This LabVIEW program was also used for controlling image acquisition.

### 2.2. Quantitative Analysis and Visualization Pipeline

Color rendering: Each raw polarization image was organized into macro pixels with 4 sub-pixels (2 × 2 configuration) carrying the information of 4 polarization states. The conventional R, G, and B intensity images were obtained by adding the sub-pixel values of each macro pixel within the respective raw images (Lower panel in Figure 1). The process converted the polarization-sensitive pixel intensity to regular pixel intensity and reduced the image size to one-quarter of the raw image. To synthesize a color image with a correct white balance, we normalized the R, G, and B images to their corresponding background intensity, which was calculated within a small region of interest in the background: a non-sample region in the transmission mode and a gray-card region in the reflection mode. A color image was then generated by synthesizing the normalized R, G, and B images. The background normalization allowed for accurate and consistent color rendering.

Quantitative polarization analysis and overlaid visualization: The raw images of the green channel were used for calculating the collagen fiber orientation and retardance map, as the circular polarizer achieved the most circularly polarized illumination around 560 nm. A higher retardance value corresponds to a higher degree of fiber alignment and higher collagen content. The retardance map was used to generate collagen content-weighted fiber orientation maps for visualization. Each raw image was split into 4 polarization images (*I*_0_, *I*_45_, *I*_90_, and *I*_135_). The collagen fiber orientation (*ϕ*) (Equation (1)) [22] and retardance (*δ*) (Equation (2)) [6] were then calculated as follows:

(1) $$\phi =\frac{1}{2}\tan^{-1}(\frac{I_{90}-I_{0}}{I_{45}-I_{135}})$$

(2) $$\delta =\sin^{-1}(\sqrt{{\left(I_{90}-I_{0}\right)}^{2}+{\left(I_{135}-I_{45}\right)}^{2}}/(I_{0}+I_{45}+I_{90}+I_{135}))$$

Simultaneous visualization of both color and collagen information (orientation and distribution) of the sample was achieved by overlaying the collagen information as a transparency layer over the color image. The local transparency of the overlaid image was set by the local retardance values. As a result, the region with stronger retardance values will show brighter collagen information and vice versa. Collagen orientation was typically displayed in the HSV color scale. However, in some cases, the predominant collagen orientations may exhibit similar colors to the appearance of stained samples. To improve the color contrast between the sample and fiber orientation, the HSV color scale may need to be circularly rotated by certain degrees, e.g., 45 degrees, for enhanced visualization. The retardance map was also visualized in green color as a surrogate of collagen distribution. Each set of color and polarization-specific images was generated using three R, G, and B images, which effectively reduced the frame rate after processing to 25 FPS. To visualize continuous tissue deformation, processed images were loaded into FIJI [23], and playback was set to 25 FPS.

### 2.3. Sample Preparation and Imaging

Microscopy imaging: We imaged two preprepared histology slides of human skin (H5, Ward’s Science, Rochester, NY, USA) and tendon fibrous connective tissue (93w3248, Ward’s Science, Rochester, NY, USA) featuring collagen in each tissue section. Both slides were previously stained. The tissue section was imaged with a 4× objective lens.

Wide-field imaging: We purchased whole chicken legs from a local grocery store. We carefully dissected the lower leg to expose tendons and the thigh to expose the sciatic nerve and blood vessels. The tendon, nerve, and vessel tissues were selected due to their high collagen content. Particularly, the nerve and vessel tissues are relevant to surgical training [24]. To demonstrate the ability of ColorPOL to quantify and track tissue deformation at high speed, we stretched and rotated the collagenous tissues (tendon, nerve, and vessel tissues) in different directions with a tweezer. The tissue deformation was captured continuously at 75 FPS. A small piece of gray card (1 cm × 1 cm) was placed within the imaging field to facilitate color calibration.

## 3. Results

**Figure 3:**
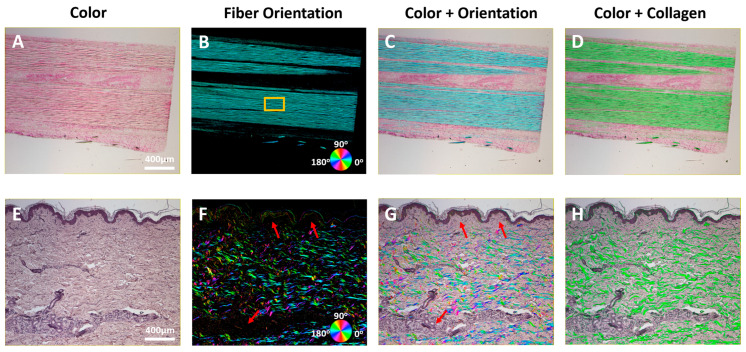
Tendon section and human skin section imaged with ColorPOL microscope with a 4× objective. For each sample, figure shows the following: (**A**,**E**) reconstructed color images; (**B**,**F**) fiber orientation maps; (**C**,**G**) merged visualization of fiber orientation map over the color image; (**D**,**H**) merged visualization of collagen content over the color image. Red arrows indicate regions with lower collagen content.

In microscopy imaging (transmission mode), we demonstrated that the true color of the tissue sections can be recovered, and the collagen content can be quantified and visualized using ColorPOL. Figure 3 shows that two representative samples, a mammalian tendon section and a human skin section, have distinct collagen organization. Both sections were previously stained, and the reconstructed color images (Figure 3A,E) faithfully show their color appearance with excellent details. The quantified collagen orientation of the tendon section clearly shows highly organized collagen fibers with a mean fiber orientation of 173.9 degrees in the region of interest (ROI) indicated with an orange box. The uniform color indicates that the collagen fibers are primarily organized in a parallel fashion, which is consistent with the known collagen organization in tendons. Merging the color and orientation information in a single image significantly improved the ability to visualize the quantitative collagen information in the context of non-collagen tissues (Figure 3C). It is worth noting that the orientation was overlaid on the color image as a transparency layer. As a result, the transparency level could be adjusted to optimize the visualization. In the human skin section, the fiber orientation map (Figure 3F) shows a highly tortuous collagen organization with a few regions showing less collagen presence, as indicated with orange arrows. Overlaying the fiber orientation map over the color image (Figure 3E) revealed that those regions were either in the epidermal region or the vascular region with lower collagen content (Figure 3G). If the collagen distribution rather than orientation is of higher importance, the collagen content can be visualized with green color for improved clarity (Figure 3D,H).

**Figure 4:**
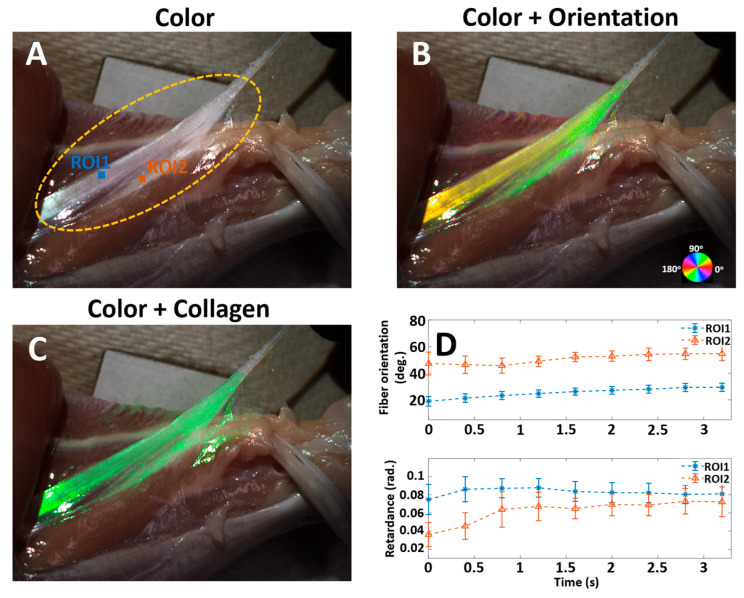
(**A**): Reconstructed color image showing a piece of tendon being stretched. The yellow oval indicates the ROI processed for visualization. (**B**): Fiber orientation map overlaid on the color image. (**C**): Collagen content overlaid on the color image. (**A**–**C**) are extracted frames at t = 1.64 s from Video S1. (**D**). Tracking of fiber orientation and retardance with two ROIs (blue and orange shown in (**A**)).

By deforming and imaging collagen content in chicken tissues, we demonstrated the ColorPOL’s high-speed imaging capability of thick tissue in reflection mode. Figure 4A shows the reconstructed color image of the dissected lower leg with a chicken tendon under stretch-induced tension (extracted frame at t = 1.64 s from Video S1). The image shows the natural colors of the muscle tissues and the tendon. The oval shape indicates the region of interest processed for visualization. Figure 4B visualizes the fiber orientation as an overlay over the color image. It is evident that the two groups of fiber merge into a single strand, as indicated by the color. The collagen overlay (Figure 4C) provides a cleaner and consistent way of visualizing the tendon content. Two ROIs (blue and orange), shown in Figure 4A, were tracked manually every 10 frames. The upper panel in Figure 4D indicates that local fiber orientation steadily increased as both extensional and rotational stretches were applied. The mean fiber orientation in the two ROIs showed a consistent 25-degree difference throughout the stretch. The retardance tracking reveals that upon the extensional stretch being applied, the collagen fibers started to uncrimp and realign, resulting in a rapid increase in retardance in the first 0.8 s. As the stretch changed from extensional to rotational around 0.8 s, as evident in Video S1, the fibers in ROI2 continued to experience low-level extensional force while the fibers in ROI1 started to experience low-level relaxation. These distinct mechanical conditions correlate well with the tracked retardance: a gradual decrease in ROI1 and a gradual increase in ROI2. Independently tracking both fiber orientation and retardance at a high speed provided valuable information for investigating the behavior of collagenous tissue under load.

**Figure 5:**
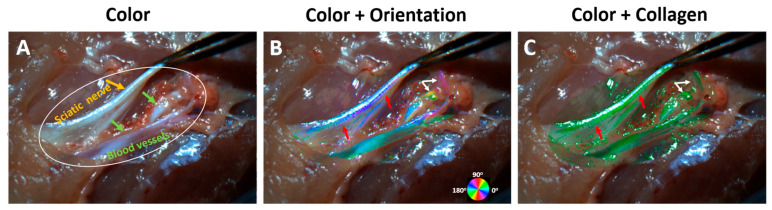
(**A**): Reconstructed color image of the dissected chicken thigh with exposed sciatic nerves (orange arrow) and blood vessels (green arrows). The orange oval indicates the region processed for visualization. (**B**): Fiber orientation overlay on the color image. Red arrows indicate collagen crimp features and white arrows indicate features with low visibility in the color image. (**C**): Collagen content overlay on the color image.

Unlike tendon tissues, sciatic nerve and blood vessel tissues have a more complex tissue composition with less collagen content [25,26,27]. As a result, the retardance values are lower, making it more challenging to separate collagen content from the non-collagenous tissues. Figure 5 shows one frame extracted from the video (Video S2), visualizing the sciatic nerves and blood vessels being pulled. Similar to the chicken tendon demonstration, the video simultaneously shows three visualization options: color (Figure 5A), orientation overlay (Figure 5B), and collagen overlay (Figure 5C). The overlay was only applied to an oval region shown in Figure 5A to improve the processing speed and highlight the region of interest. The fiber orientation overlay (Figure 5B) not only shows the quantitative local fiber orientation, but it also makes tissue features such as crimp more visible (red arrows in Figure 5B), which were otherwise almost invisible in the color image. It is worth noting that due to the complexity of collagen organization in these tissues, the quantified local collagen orientation may not align with the apparent longitudinal shape of the nerve and the vessel [25,26]. Overlaying the collagen content (Figure 5C) over the color tissue image further enhanced the contrast and visibility of the nerve and the vessels. Due to specular surface reflections, sparse non-collagen regions are also visible and shown in green. These non-collagen regions could be effectively suppressed with an improved segmentation.

## 4. Discussion

In this study, we demonstrated that ColorPOL is a robust imaging technique that can acquire both color and collagen-specific information at high speed. ColorPOL can be used to image both thin tissue sections in transmission mode and thick tissues in reflection mode. Compared to existing imaging technologies, ColorPOL exhibits multiple advantages. ColorPOL uses only one camera to acquire images needed for generating color and collagen-specific images, which eliminates the need for image registration typically required with dual-camera systems. Furthermore, the single-camera configuration effectively reduces the system cost and complexity, potentially making the adoption of ColorPOL more accessible.

Polychromatic/instant polarization microscopy can generate vivid colors of collagen optically without the need for post-processing, which is particularly suitable for direct visualization and for imaging tissue dynamics. The color represents the orientation and density of the collagen. However, polychromatic/instant polarization microscopy has not demonstrated its applicability to thick tissues in reflection mode. This is likely due to strong optical scattering in thick tissues, which may render this imaging mechanism less effective. Furthermore, while color-based quantification (e.g., fiber orientation) has been demonstrated with mostly colorless samples, it is challenging to derive accurate quantification from the samples that have been previously stained. The dye present in the tissue will introduce additional absorption, thus altering the color and rendering the color-based quantification less reliable. Finally, with a single-shot color camera in polychromatic/instant polarization microscopy, the color of collagen from polarization is blended with the native color of the sample in the acquired images. In contrast, with ColorPOL, the information sources on fiber orientation, collagen density, and distribution are overlaid on the true color image. This provides the user the flexibility to adjust the saturation and transparency of the overlay to selectively highlight either the native or the derived information.

There are color polarization-sensitive cameras available that can acquire both types of information with a single shot. However, compared to its monochromatic counterpart, the color information is acquired by adding a Bayer filter mosaic on top of macro pixels (2 × 2 configuration), resulting in even larger macro pixels (4 × 4 configuration). Color information is recovered through de-mosaicking, which potentially leads to false color artifacts and reduced image resolution, which are particularly undesirable in microscopy. ColorPOL, on the other hand, acquires RGB information in full resolution and eliminates color interpolation, which generates more accurate color and preserves the finer structural information of the sample. Moreover, the Bayer filter mosaic used in the color polarization camera is preconfigured and has a specific spectral response, which limits such cameras to true color imaging. In our study, the true color information was acquired by switching the RGB illumination rapidly during imaging. The spectral bands of illumination (UV, visible, and NIR) can be specifically selected and optimized to enable additional imaging capabilities, including narrowband imaging, fluorescence imaging, and multispectral imaging, potentially resulting in enhanced tissue contrast and visibility.

The ColorPOL imaging system acquires images at 75 FPS, which is equivalent to 25 FPS after processing. While the frame rate is not high enough for certain imaging purposes, such as imaging fast tissue dynamics, it is sufficient for visual inspection and navigational purposes, with potential value in applications such as image-guided surgery. Due to the cyclic nature of the R, G, and B images, it is feasible to further improve the visual smoothness by generating color images using the rolling processing technique, similar to the technique reported in [22]. Briefly, the rolling image processing updates and processes a group of three images by removing the first channel image and adding the next channel image in the sequence to the group, which will result in visualizations at an effective 75 FPS. The color image is synthesized with three images spanning 40 ms. If significant motion occurs within 40 ms, color fringes may appear due to the imperfect alignment of the R, G, and B images. However, collagen information is less susceptible to motion artifacts because it is based on a single green image. It is important to note that the imaging speed of the ColorPOL system is limited by the maximum frame rate of the camera, while the illumination system can operate at a much higher speed.

Real-time image processing and visualization are critical to applications such as image-guided intervention. While our current system has not been implemented for real-time visualization, the image processing pipeline is highly efficient and can generate results for visualization approximately every 0.25 s using an Intel i7-11850H CPU (Intel, Santa Clara, CA, USA). A faster processor could further reduce the processing time. The real-time imaging capability will be investigated in future studies. For transmission imaging, we implemented ColorPOL on an upright microscope. A similar implementation is expected to be feasible on an inverted microscope as well. A 4× objective lens was used for imaging tissue slides. Objective lenses with a higher magnification can be used if greater spatial resolution is needed.

## 5. Conclusions

In this study, we demonstrated that ColorPOL is a robust and easy-to-implement polarized light imaging technology that can capture and visualize both color and collagen data at a high frame rate. ColorPOL can be implemented in both transmission and reflection modes, facilitating the characterization of thin and thick tissues. While the visualization was generated offline in the current study, our streamlined data processing pipeline can potentially be implemented for real-time applications.

## Data Availability

The data that support the findings of this study are available from the corresponding author upon reasonable request.

## Supplementary Materials

The following supporting information can be downloaded at https://www.mdpi.com/article/10.3390/bioengineering12070720/s1. Video S1: Tendon stretching; Video S2: Nerve and blood vessel stretching.

## References

[1] Franchi M., Trirè A., Quaranta M., Orsini E., Ottani V. (2007). Collagen structure of tendon relates to function. Sci. World J..

[2] Tedder M.E., Liao J., Weed B., Stabler C., Zhang H., Simionescu A., Simionescu D.T. (2009). Stabilized collagen scaffolds for heart valve tissue engineering. Tissue Eng. Part A.

[3] Reilly D.M., Lozano J. (2021). Skin collagen through the lifestages: Importance for skin health and beauty. Plast. Aesthetic Res..

[4] Meek K.M., Boote C. (2004). The organization of collagen in the corneal stroma. Exp. Eye Res..

[5] Fratzl P. (2008). Collagen: Structure and mechanics, an introduction. Collagen: Structure and Mechanics.

[6] Jan N.-J., Grimm J.L., Tran H., Lathrop K.L., Wollstein G., Bilonick R.A., Ishikawa H., Kagemann L., Schuman J.S., Sigal I.A. (2015). Polarization microscopy for characterizing fiber orientation of ocular tissues. Biomed. Opt. Express.

[7] Yang B., Jan N.J., Brazile B., Voorhees A., Lathrop K.L., Sigal I.A. (2018). Polarized light microscopy for 3-dimensional mapping of collagen fiber architecture in ocular tissues. J. Biophotonics.

[8] Bromage T.G., Goldman H.M., McFarlin S.C., Warshaw J., Boyde A., Riggs C.M. (2003). Circularly polarized light standards for investigations of collagen fiber orientation in bone. Anat. Rec. Part B New Anat. Off. Publ. Am. Assoc. Anat..

[9] Rieppo J., Hallikainen J., Jurvelin J.S., Kiviranta I., Helminen H.J., Hyttinen M.M. (2008). Practical considerations in the use of polarized light microscopy in the analysis of the collagen network in articular cartilage. Microsc. Res. Tech..

[10] Pircher M., Götzinger E., Leitgeb R., Sattmann H., Findl O., Hitzenberger C.K. (2004). Imaging of polarization properties of human retina in vivo with phase resolved transversal PS-OCT. Opt. Express.

[11] Giattina S.D., Courtney B.K., Herz P.R., Harman M., Shortkroff S., Stamper D.L., Liu B., Fujimoto J.G., Brezinski M.E. (2006). Assessment of coronary plaque collagen with polarization sensitive optical coherence tomography (PS-OCT). Int. J. Cardiol..

[12] Mansfield J.C., Mandalia V., Toms A., Winlove C.P., Brasselet S. (2019). Collagen reorganization in cartilage under strain probed by polarization sensitive second harmonic generation microscopy. J. R. Soc. Interface.

[13] Stoller P., Reiser K.M., Celliers P.M., Rubenchik A.M. (2002). Polarization-modulated second harmonic generation in collagen. Biophys. J..

[14] Cha J., Broch A., Mudge S., Kim K., Namgoong J.-M., Oh E., Kim P. (2018). Real-time, label-free, intraoperative visualization of peripheral nerves and micro-vasculatures using multimodal optical imaging techniques. Biomed. Opt. Express.

[15] Liu Y., Dong Y., Si L., Meng R., Dong Y., Ma H. (2021). Comparison between image texture and polarization features in histopathology. Biomed. Opt. Express.

[16] Ning B., Kim W.W., Katz I., Park C.H., Sandler A.D., Cha J. (2021). Improved nerve visualization in head and neck surgery using mueller polarimetric imaging: Preclinical feasibility study in a swine model. Lasers Surg. Med..

[17] Shribak M. (2015). Polychromatic polarization microscope: Bringing colors to a colorless world. Sci. Rep..

[18] Yang B., Lee P.Y., Hua Y., Brazile B., Waxman S., Ji F., Zhu Z., Sigal I.A. (2021). Instant polarized light microscopy for imaging collagen microarchitecture and dynamics. J. Biophotonics.

[19] Lee P.-Y., Schilpp H., Naylor N., Watkins S.C., Yang B., Sigal I.A. (2023). Instant polarized light microscopy pi (IPOLπ) for quantitative imaging of collagen architecture and dynamics in ocular tissues. Opt. Lasers Eng..

[20] Keikhosravi A., Shribak M., Conklin M.W., Liu Y., Li B., Loeffler A., Levenson R.M., Eliceiri K.W. (2021). Real-time polarization microscopy of fibrillar collagen in histopathology. Sci. Rep..

[21] Rebhan D., Rosenberger M., Notni G. Principle investigations on polarization image sensors. Proceedings of the Photonics and Education in Measurement Science 2019.

[22] Ingram G., Lee P.-Y., Bernarding B., Sigal I.A., Yang B. Real-time structured polarized light imaging of dynamics of thick collagenous tissues. Proceedings of the Emerging Digital Micromirror Device Based Systems and Applications XIII.

[23] Schindelin J., Arganda-Carreras I., Frise E., Kaynig V., Longair M., Pietzsch T., Preibisch S., Rueden C., Saalfeld S., Schmid B. (2012). Fiji: An open-source platform for biological-image analysis. Nat. Methods.

[24] Chong A.K., Le L.A.T., Lahiri A., Yusoff K., Yip G.W., Pan F., Teo W., Liao J.C., Lim J.X. (2023). Surgical anatomy and exercises using the chicken thigh sciatic nerve for microsurgery training. J. Hand Microsurg..

[25] Ushiki T., Ide C. (1990). Three-dimensional organization of the collagen fibrils in the rat sciatic nerve as revealed by transmission-and scanning electron microscopy. Cell Tissue Res..

[26] Eble J.A., Niland S. (2009). The extracellular matrix of blood vessels. Curr. Pharm. Des..

[27] Fertala J., Rivlin M., Wang M.L., Beredjiklian P.K., Steplewski A., Fertala A. (2020). Collagen-rich deposit formation in the sciatic nerve after injury and surgical repair: A study of collagen-producing cells in a rabbit model. Brain Behav..

